# Is there an association between superoxide dismutase gene polymorphisms, antioxidants in oxidative stress pathway, and oral health-related quality of life after root canal treatment?

**DOI:** 10.2340/aos.v84.43426

**Published:** 2025-04-22

**Authors:** Ludmila da Silva Guimarães, Erlange Andrade Borges da Silva, Juliana Miranda Bonelli, Heitor Ganier Ribeiro, Alice Corrêa Silva-Sousa, Manoel Damião Sousa-Neto, Erika Calvano Küchler, Lívia Azeredo Alves Antunes, Fernanda Signorelli Calazans, Leonardo Santos Antunes

**Affiliations:** a Postgraduate Program, School of Dentistry, Fluminense Federal University, Niterói, Rio de Janeiro, Brazil; b Postgraduate Program, School of Dentistry, Fluminense Federal University, Nova Friburgo, Rio de Janeiro, Brazil; c Specific Formation Department, School of Dentistry of Nova Friburgo, Fluminense Federal University, Nova Friburgo, Rio de Janeiro, Brazil; d Restorative Dentistry Department, School of Dentistry of Ribeirão Preto, University of São Paulo, Ribeirão Preto, São Paulo, Brazil; e Department of Orthodontics, University Hospital Bonn, Bonn, Germany

**Keywords:** Genetic polymorphism, oxidative stress, superoxide dismutase, oral health-related quality of life, periapical periodontitis

## Abstract

**Objective:**

To evaluate the association between genetic polymorphisms in *SOD2* and *SOD3* and oral health-related quality of life (OHRQoL).

**Material and methods:**

The cohort study included 109 participants, all of whom underwent root canal treatment on a single-rooted maxillary or mandibular tooth with asymptomatic periapical periodontitis. The OHRQoL was assessed using the Oral Health Impact Profile-14 at three intervals: prior to root canal treatment (T0), 7 (T7), and 30 (T30) days post-treatment. Genomic DNA was extracted from buccal cells for genotyping of polymorphisms in *SOD2* (rs5746136, rs4880, and rs10370) and *SOD3* (rs2855262 and rs13306703) using real-time PCR. Both Univariate and Multivariate Poisson Regression analyses were conducted, with *p* < 0.05 indicating statistical significance.

**Results:**

The rs2855262 polymorphism in the *SOD3* gene showed a significant difference in the functional limitation domain in both codominant (*p* = 0.037) and recessive (*p* = 0.040) models. The rs13306703 polymorphism in *SOD3* demonstrated a significant difference in physical pain [in codominant (*p* = 0.001) and recessive (*p* < 0.001) models], psychological discomfort [in codominant (*p* = 0.002) and recessive (*p* = 0.002) models], handicap [in codominant (*p* = 0.011) and dominant (*p* = 0.015) models], and total score [in codominant (*p* = 0.011) and recessive (*p* = 0.007) models]. In the Multivariate Poisson Regression analysis, *SOD2* (rs5746136) was associated with the psychological disability domain [in codominant (*p* = 0.049) and recessive (*p* = 0.040) models] and *SOD3* (rs13306703), in the handicap domain [in codominant (*p* = 0.028) and dominant (*p* = 0.037) models].

**Conclusions:**

Genetic polymorphisms in *SOD2* and *SOD3* genes can influence the OHRQoL response in patients with asymptomatic periapical periodontitis.

## Introduction

Oral health, an integral component of overall health, includes the ability to perform functions such as speaking, smiling, smelling, tasting, touching, chewing, swallowing, and expressing emotions facially with confidence, free from pain, discomfort, and craniofacial complex diseases [1]. Oral conditions can profoundly impact an individual’s perceived quality of life [2]. Consequently, findings derived from patients’ self-perception of oral health and oral health-related quality of life (OHRQoL) offer a valuable opportunity to supplement clinical data with the patient’s viewpoint. This approach is crucial in clinical dental practice, dental education, and research, facilitating improved clinical decision-making [2, 3].

Single nucleotide polymorphisms (SNPs), the most frequent form of gene polymorphisms, involve the substitution or deletion of a single base with a minor allele frequency of >1%. SNPs in protein-encoding genes can influence a phenotype by changing the structure and function of the protein or the level of the encoded protein [4]. In this theme, the International and Interdisciplinary Consortium for Genetics and Quality of Life Research, the GENEQOL, was an initiative created to investigate the genetic disposition of patient-reported quality-of-life outcomes [5]. The Consortium aimed to create a comprehensive list of potential biological pathways, genes, and genetic variants associated with quality of life, by reviewing current genetic knowledge [5, 6]. Sloan and Zhao [7] pioneered the investigation of a direct connection between genetic polymorphisms and cancer patients’ quality of life through a clinical trial conducted by the North Central Cancer Treatment Group. In the field of dentistry, few studies have already established a correlation between OHRQoL and genetic polymorphisms [8–12]. However, previous studies demonstrate that endodontic factors affect OHRQoL [13–16].

Periapical periodontitis is a chronic inflammatory disorder of periradicular tissues caused by etiological agents of endodontic origin [17], and half of the adult population worldwide has at least one tooth with apical periodontitis [18]. It is viewed as a dynamic encounter between microbial factors and host defenses at the interface between infected radicular pulp and the periodontal ligament that results in local inflammation, resorption of hard tissues, destruction of other periapical tissues, and eventual formation of various histopathological categories of apical periodontitis [19]. Elevated levels of inflammatory mediators are observed in the blood of individuals with periapical periodontitis [20]. These mediators are activated by reactive oxygen species (ROS), triggering an innate immune response [21].

ROS and antioxidant mechanisms interact to maintain physiological balance. ROS serve as a crucial host defense mechanism against invading pathogens [22]. Superoxide dismutases (SODs) are the first and most important line of antioxidant enzyme defense systems against ROS [23]. Oxidative stress arises when ROS overproduction is not counterbalanced by sufficient antioxidant levels [24]. This imbalance can be triggered by physiological events such as aging and bone loss [25], as well as pathological events related to the production of inflammatory cytokines involved in many pathological processes, exposure to exogenous and endogenous toxins, radiation, and drug therapies [24, 26]. Oxidative stress can induce local periapical tissue damage [21, 22] and contribute to chronic inflammatory disorders, including atherosclerosis, arthritis, and cancer [22, 26]. Moreover, oxidative stress is implicated in various mental disorders, encompassing depression, anxiety disorders, schizophrenia, and bipolar disorder [27]. Changes in SOD activity have been associated with psychological disorders such as depression and generalized anxiety disorder [28, 29]. According to Maes et al. [28], the activation of inflammatory, oxidative, and nitrosative stress pathways is a crucial pathophysiological factor in these disorders.

To date, no study has assessed the relationship between *SOD* gene polymorphisms and OHRQoL in patients with asymptomatic periapical periodontitis. Inflammatory pathways and individual genetic factors can influence oral changes in quality of life, either positively or negatively [6, 30]. As a result, gaining new insights into the influence of genetic variations on OHRQoL is crucial for identifying potential indicators within the field of dentistry [11], particularly in prevalent and significant oral disorders such as periapical periodontitis [18].

Based on the aforementioned information, these genetic polymorphisms were selected owing to their potential clinical relevance to inflammation and the previous findings suggesting that these inflammatory pathways could influence the quality-of-life domain [6]. Therefore, this study aimed to investigate the association between *SOD2* and *SOD3* genetic polymorphisms and OHRQoL in individuals undergoing root canal treatment. The hypothesis tested was that these genetic polymorphisms modulate the impact of OHRQoL in patients with asymptomatic periapical periodontitis undergoing root canal treatment. Understanding the interplay between genetic and environmental components in patient-reported quality-of-life provides opportunities for innovative person-centered approaches, improving patient care, overall well-being and treatment outcomes.

## Materials and methods

### Study design and ethical aspects

This cohort study adhered to the checklist provided by the Strengthening the Reporting of Genetic Association Studies (STREGA) statement [31]. Prior approval was obtained from the Human Ethics Committee of the Fluminense Federal University/Health Institute of Nova Friburgo (approval number: 5.545.749). All participants in the research provided their signatures on the informed consent form after being apprised of the potential risks and benefits associated with the treatment.

### Participants selection

The researchers examined and selected the sample based on the following inclusion and exclusion criteria, before conducting the laboratory analysis. The sample included 109 participants, all aged >18 years, who received root canal treatment on single-rooted maxillary or mandibular teeth, with a single-canal, necrotic pulp associated with asymptomatic periapical periodontitis. The treatments were conducted at the Fluminense Federal University/Health Institute of Nova Friburgo, Rio de Janeiro, Brazil, between July 2022 and July 2023. The exclusion criteria were as follows: patients experiencing preoperative pain or edema; those who were pregnant or lactating; individuals with an allergy to sodium hypochlorite (NaOCl) or ibuprofen; patients who had taken antibiotics within the past 30 days [32]; and those who had used analgesics or anti-inflammatories within the last 24 h or required antibiotic pre-medication for dental treatment. Additionally, teeth where the patency of the foramen could not be determined, cases requiring root canal retreatment, and vital teeth or teeth with necrosis but without periapical periodontitis were also excluded.

A sample size calculation for a Poisson regression analysis was conducted to assess the impact of a dependent variable of counts (OHRQoL impact ‘yes’ or ‘no’) on a nominal independent variable, following the methodology outlined by Signorini [33]. This test was performed using PASS 2021 software (NCSS, LLC, Kaysville, Utah, USA).The calculation utilized several parameters derived from a Poisson regression study by Meyfarth et al. [34], which examined the effect of the rs6553010 (MTNR1A) SNP on pain rates following root canal treatment. These parameters included a baseline rate of 0.33 (the rate of cases), a mean exposure time of 1.00 (indicating variation in response rates over time), a response rate ratio of 0.24, and a covariate proportion (minor allele frequency) of 0.24. The sample size calculation concluded that a total of 101 participants is enough to achieve 80% power to detect a significant response rate at a 0.05 significance level. Considering a 10% dropout rate (i.e., the percentage of subjects expected to be lost during the study), we planned to recruit 111 participants for this study.

### Data collection

#### Treatment protocol

A single specialist (L.S.G), with 10 years of experience, performed the clinical procedures in a single session, adhering to the protocol standardized in the Guimaraes et al. [35] study.

After local anesthesia with 2% lidocaine with 1:100,000 epinephrine (DFL Indústria e Comércio Ltda, Taquara, Rio de Janeiro, Brazil), endodontic access was performed with a sterile diamond bur (KG Sorensen, Cotia, São Paulo, Brazil). The rubber dam was placed and disinfected with 2.5% NaOCl (Fórmula & Ação, São Paulo, Brazil). Each tooth was irrigated with 15 mL of 2.5% NaOCl (Fórmula & Ação, São Paulo, Brazil) [36].

The working length was determined at the ‘00’ mark [32] using the RomiApex A-15 apex locator (Romidan, Kiryat Ono, Israel). Patency was maintained with a size 10 K-file (Dentsply Sirona, York, Pennsylvania, USA) throughout the instrumentation phase with a reciprocation instrument.

The root canal obturation process involved a 5-min irrigation with 17% EDTA, followed by neutralization using 2 mL of saline solution. The root canal was then dried using sterile absorbent paper cones. Gutta-percha and MTA Fillapex sealer (Angelus, Londrina, Paraná, Brazil) were applied using lateral condensation. Subsequently, a definitive restoration was carried out, and the occlusion was examined and adjusted as necessary.

#### OHRQoL assessment

The OHRQoL assessment in patients undergoing root canal treatment was conducted using the Brazilian short-form of the Oral Health Impact Profile (OHIP-14) [37]. This was done through face-to-face interviews at three distinct intervals: prior to the root canal treatment (T0), 7 days post-treatment (T7), and 30 days post-treatment (T30). The interviews were carried out by a professional who had received prior training but did not participate in the clinical procedure (E.A.B.S).

The questionnaire comprises 14 items, conceptually divided into seven dimensions: functional limitation, physical pain, psychological discomfort, physical disability, psychological disability, social disability, and handicap. The responses were based on a five-point Likert scale: 0 = never; 1 = hardly ever; 2 = occasionally; 3 = fairly often; and 4 = very often. OHIP-14 scores were computed using the additive method, with the assessment scale ranging from 0 to 56. Higher scores indicated a greater negative impact on OHRQoL [37]. Based on the OHIP-14 scores, the impact on OHRQoL was classified as either without impact (0) or with impact (1–56).

#### Other variables

The multivariate analysis was adjusted to include the independent variables of gender (woman and man), ethnicity, dental arch (mandible or maxilla), medication, and the size of periapical periodontitis.

A single operator (E.A.B.S) determined the area of periapical periodontitis in mm^2^ using the specific tools of the ImageJ/Fiji 1.46 software (http://imagej.nih.gov/ij/). This was accomplished through an analysis of the initial radiograph.

#### Deoxyribonucleic acid sample extraction and genotyping

All participating individuals provided deoxyribonucleic acid (DNA) samples, which were extracted from their buccal cells following a protocol established by Küchler et al. [38]. The collection of buccal cells involved a 60-s mouth rinse with 15 mL of saline, followed by expectoration into a 50 mL propylene tube. Only DNA samples with an A260 nm/A280 nm ratio of less than 1.8 were utilized, as per Küchler et al. [38] and Aidar and Line [39].

The genotyping of polymorphisms in *SOD2* (rs5746136, rs4880, and rs10370) and *SOD3* (rs2855262 and rs13306703) genes was conducted using real-time PCR with the TaqMan method (Applied Biosystems, Foster City, California, USA). The characteristics of the genetic polymorphisms under study are detailed in Table 1. The Hardy–Weinberg equilibrium was assessed, and only those results conforming to the Hardy–Weinberg equilibrium were subjected to further analysis [10].

**Table 1:** Details on the genetic markers’ studied.

Gene (genetic polymorphism)	Position	Functional consequence	Alleles	Frequency of the polymorphic genotype (homozyguous) in the studied population (*n*)
*SOD3* (rs2855262)	Chr.4: 24800354 on GRCh38	UTR3, Transition Substitution, Intragenic	C/T	CC = 0.19
*SOD3* (rs13306703)	Chr.4: 24793891 on GRCh38	Intergenic/Unknown, Transition Substitution, Intragenic	C/T	TT = 0.03
*SOD2* (rs5746136)	Chr.6: 159682052 on GRCh38	Intron, UTR 3, Transition Substitution, Intragenic	C/T	TT = 0.06
*SOD2* (rs4880)	Chr.6: 159692840 on GRCh38	Missense Mutation, Transition Substitution, UTR 5, Intragenic	A/G	GG = 0.25
*SOD2* (rs10370)	Chr.6: 159680500 on GRCh38	Intron, Transversion Substitution, UTR 3, Intragenic	T/G	GG = 0.04

Notes: Obtained from databases: http://www.thermofisher.com, http://www.ncbi.nlm.nih.gov and http://genome.ucsc.edu.

All analyses were conducted in the Fluminense Federal University laboratory. The laboratory examiners were blinded to genotyping. Each plate included two negative controls. To ensure internal consistency, 10% of all samples were randomly retested. The results demonstrated a 100% consistency rate.

### Statistical analysis

The Generalized Estimating Equations (GEE) were utilized to examine the distribution of participants, both with and without impact on OHRQoL, across different genotypes and experimental times. The choice of GEE allows for modeling the correlation between repeated measurements within the same subject over time. Univariate and Multivariate Poisson Regression were employed. The OHRQoL scores were used as a dichotomized and dependent variable in the Poisson regression model. Any genetic polymorphism that approached a *p*-value of less than 0.05 in the Univariate analysis was further analyzed in the Multivariate Poisson Regression, with adjustments made for factors associated with each domain. A previous univariate analysis (utilizing Pearson chi-square or Fisher exact test) was conducted to identify potential confounding factors associated with the frequency of oral health impacts experienced by this sample. In this way, the Poisson regression models were adjusted only to variables that were previously associated with the OHRQoL scores. Prevalence Ratio (PR) with 95% Confidence interval (CI) were obtained to measure the effect size.

All analyses were conducted using IBM SPSS version 25.0 (IBM Corp, Armonk, New York, USA), with a *p*-value of less than 0.05 indicating statistical significance. The Hardy–Weinberg equilibrium was evaluated using Pearson’s Chi-square test.

## Results

**Figure 1:**
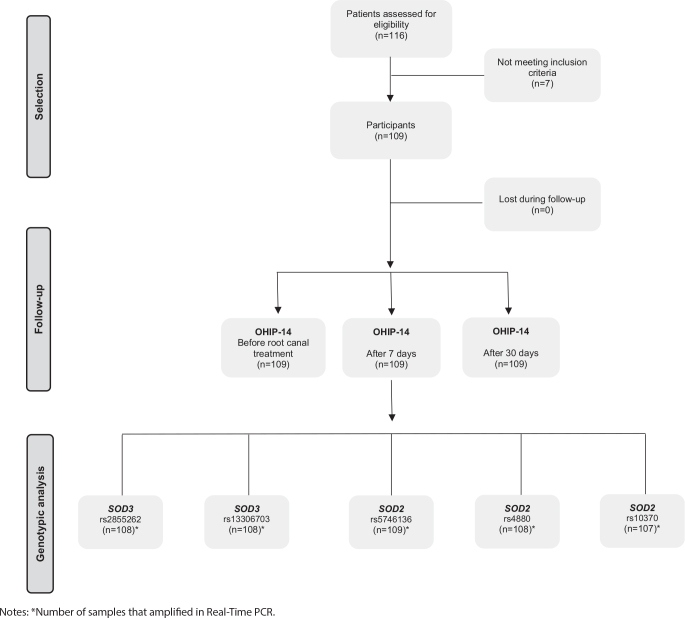
Study design flowchart.

A total of 116 individuals underwent eligibility assessment. Seven of these individuals failed to meet the inclusion criteria, resulting in 109 participants being included in the study. There were no instances of patient loss during the follow-up period (Figure 1).

Out of 109 participants, 71 (65.1%) were women and 38 (34.9%) were men. The participants’ ages varied from 18 to 70 years, with a mean age of 40.4 years (standard deviation [SD] = 13.1).

According to the participants’ perception of their OHRQoL, on the total score domain of the OHIP-14 instrument, two participants perceived no negative impact on their OHRQoL prior to root canal treatment, while 107 reported some impact. Seven days post-procedure, 48 participants reported no impact on their OHRQoL, contrasted with 61 who experienced a negative impact. After 30 days, the number of participants reporting no impact increased to 59, with 50 still experiencing a negative impact.

The frequencies of *SOD2* (rs5746136, rs4880, and rs10370) and *SOD3* (rs2855262 and rs13306703) were in Hardy-Weinberg equilibrium (data not shown).

Table 2 illustrates the influence of genetic polymorphisms on the total score domain prior to and 7 and 30 days following root canal treatment. A significant difference in the total score was only observed in the rs13306703 polymorphism of the *SOD3* gene, both in codominant (*p* = 0.011) and recessive (*p* = 0.007) models.

**Table 2:** Impact of genetic polymorphisms on the total score of OHIP-14 before, 7 and 30 days after root canal treatment.

Total score																
Gene	Genetic polymorphism	Model	Genotype	Before treatment				After 7 days				After 30 days				*P*
				No impact		Impact		No impact		Impact		No impact		Impact		
				*N*	%	*N*	%	*N*	%	*N*	%	*N*	%	*N*	%	
*SOD3*	rs2855262	Co-Dominant	TT	1	50.00	30	28.30	14	29.79	17	28.33	15	25.00	16	32.65	Reference
			CT	0	0.00	56	52.83	23	48.94	33	55.00	34	56.67	22	44.90	0.795
			CC	1	50.00	20	18.87	10	21.28	10	16.67	11	18.33	11	22.45	0.491
		Dominant	TT	1	50.00	30	28.30	14	29.79	17	28.33	15	25.00	16	32.65	Reference
			CT + CC	1	50.00	76	71.70	33	70.21	43	71.67	45	75.00	33	67.35	0.948
		Recessive	TT + CT	1	50.00	86	81.13	37	78.72	50	83.33	49	81.67	38	77.55	Reference
			CC	1	50.00	20	18.87	10	21.28	10	16.67	11	18.33	11	22.45	0.346
	rs13306703	Co-Dominant	CC	1	50.00	81	76.42	36	76.60	45	75.00	46	76.67	37	75.51	Reference
			CT	1	50.00	22	20.75	11	23.40	12	20.00	12	20.00	11	22.45	0.548
			TT	0	0.00	3	2.83	0	0.00	3	5.00	2	3.33	1	2.04	0.011*
		Dominant	CC	1	50.00	81	76.42	36	76.60	45	75.00	46	76.67	37	75.51	Reference
			CT + TT	1	50.00	25	23.58	11	23.40	15	25.00	14	23.33	12	24.49	0.850
		Recessive	CC + CT	2	100.00	103	97.17	47	100.00	57	95.00	58	96.67	48	97.96	Reference
			TT	0	0.00	3	2.83	0	0.00	3	5.00	2	3.33	1	2.04	0.007*
	rs5746136	Co-Dominant	CC	0	0.00	60	56.07	24	51.06	36	59.02	31	51.67	29	58.00	Reference
			CT	1	50.00	42	39.25	21	44.68	21	34.43	25	41.67	19	38.00	0.303
			TT	1	50.00	5	4.67	2	4.26	4	6.56	4	6.67	2	4.00	0.889
		Dominant	CC	0	0.00	60	56.07	24	51.06	36	59.02	31	51.67	29	58.00	Reference
			CT + TT	2	100.00	47	43.93	23	48.94	25	40.98	29	48.33	21	42.00	0.333
		Recessive	CC + CT	1	50.00	102	95.33	45	95.74	57	93.44	56	93.33	48	96.00	Reference
			TT	1	50.00	5	4.67	2	4.26	4	6.56	4	6.67	2	4.00	0.983
*SOD2*	rs4880	Co-Dominant	AA	1	50.00	32	30.19	12	25.53	21	35.00	20	33.33	13	26.53	Reference
			AG	1	50.00	48	45.28	24	51.06	24	40.00	26	43.33	24	48.98	0.207
			GG	0	0.00	26	24.53	11	23.40	15	25.00	14	23.33	12	24.49	0.415
		Dominant	AA	1	50.00	32	30.19	12	25.53	21	35.00	20	33.33	13	26.53	Reference
			AG + GG	1	50.00	74	69.81	35	74.47	39	65.00	40	66.67	36	73.47	0.205
		Recessive	AA + AG	2	100.00	80	75.47	36	76.60	45	75.00	46	76.67	37	75.51	Reference
			GG	0	0.00	26	24.53	11	23.40	15	25.00	14	23.33	12	24.49	0.843
	rs10370	Co-Dominant	TT	0	0.00	69	65.71	28	59.57	41	69.49	38	64.41	31	63.27	Reference
			TG	1	50.00	33	31.43	17	36.17	16	27.12	18	30.51	17	34.69	0.365
			GG	1	50.00	3	2.86	2	4.26	2	3.39	3	5.08	1	2.04	0.665
		Dominant	TT	0	0.00	69	65.71	28	59.57	41	69.49	38	64.41	31	63.27	Reference
			TG + GG	2	100.00	36	34.29	19	40.43	18	30.51	21	35.59	18	36.73	0.326
		Recessive	TT + TG	1	50.00	102	97.14	45	95.74	57	96.61	56	94.92	48	97.96	Reference
			GG	1	50.00	3	2.86	2	4.26	2	3.39	3	5.08	1	2.04	0.721

SNP: single nucleotide polymorphisms.

Notes: Univariate Poisson Regression by Generalized Estimating Equations was performed to obtain *p*-value. # means that some convergence criteria were not met.

* means p < 0.05.

In the Multivariate Poisson Regression analysis using GEE, the *SOD2* polymorphism (rs5746136) was associated with the psychological disability domain [in codominant model PR = 0.43 (95% CI: 0.18–0.99), (*p* = 0.049) and recessive model PR = 0.41 (95% CI: 0.18–0.96), (*p* = 0.040)]. Similarly, the *SOD3* gene polymorphism (rs13306703) also influenced the handicap domain [in codominant model PR = 1.61 (95% CI: 1.05–2.48), (*p* = 0.028) and dominant model PR = 1.58 (95% CI: 1.02–2.43), (*p* = 0.037)] (Table 3).

**Table 3:** Multivariate Poisson regression by generalized estimating equations analysis.

Domain	Gene	Genetic polymorphism	Model	Reference	Genotype	Prevalence ratio (CI 95)	*P*	Adjusted by
Functional limitation	*SOD3*	rs2855262	Co-dominant	TT	CT	0.88 (0.50 – 1.53)	0.663	Gender
					CC	0.37 (0.14 – 1.00)	0.052	
			Recessive	TT + CT	CT	0.41 (0.16 – 1.02)	0.055	
		rs13306703	Co-dominant	CC	CT	1.05 (0.61 – 1.81)	0.853	
					TT	2.02 (0.75 – 5.43)	0.163	
			Recessive	CC + CT	CT	2.00 (0.75 – 5.29)	0.163	
	*SOD2*	rs4880	Recessive	AA + AG	AG	1.39 (0.83 – 2.35)	0.208	
Physical pain	*SOD3*	rs13306703	Co-dominant	CC	CT	0.90 (0.69 – 1.17)	0.446	Gender medication
					TT	1.31 (0.81 – 2.12)	0.263	
			Recessive	CC + CT	CT	1.33 (0.81 – 2.17)	0.246	
	*SOD2*	rs4880	Co-dominant	AA	AG	0.81 (0.65 – 1.01)	0.073	
					GG	0.81 (0.63 – 1.04)	0.107	
Psychological discomfort	*SOD3*	rs13306703	Co-dominant	CC	CT	1.01 (0.78 – 1.33)	0.892	Medication arch position
					TT	1.25 (0.82 – 1.90)	0.282	
			Recessive	CC + CT	TT	1.25 (0.83 – 1.87)	0.281	
Psychological disability	*SOD2*	rs5746136	Co-dominant	CC	CT	1.06 (0.79 – 1.42)	0.658	Periapical periodontitis size
					TT	0.43 (0.18 – 0.99)	0.049*	
			Recessive	CC + CT	TT	0.41 (0.18 – 0.96)	0.040*	
Handicap	*SOD3*	rs13306703	Co-dominant	CC	CT	1.61 (1.05 – 2.48)	0.028*	Ethnicity
					TT	1.28 (0.24 – 6.70)	0.765	
			Dominant	CC	CT + TT	1.58 (1.02 – 2.43)	0.037*	
Total score	*SOD3*	rs13306703	Co-dominant	CC	CT	0.92 (0.74 – 1.15)	0.501	Medication arch position
					TT	1.15 (0.77 – 1.72)	0.483	
			Recessive	CC + CT	TT	1.17 (0.78 – 1.76)	0.434	

CI: confidence interval; SNP: single nucleotide polymorphisms.

Notes: The analysis was performed with each genotype individually adjusted by factors indicated in the last column.

* means *p* < 0.05.

The associations between genotypes and each domain of the OHIP-14, as per the three different models (co-dominant, recessive, and dominant) are detailed in Supplementary Tables 1–7(https://osf.io/wer2d/files/osfstorage/64d6a11f70dc990068d49c85). The analysis of the rs2855262 polymorphism in the *SOD3* gene indicated significant differences in functional limitation in both the co-dominant (*p* = 0.037) and recessive (*p* = 0.040) models. In relation to the rs13306703 polymorphism in the same gene, significant differences were observed in the following domains: physical pain in both the co-dominant (*p* = 0.001) and recessive (*p* < 0.001) models; psychological discomfort in the co-dominant (*p* = 0.002) and recessive (*p* = 0.002) models; and handicap in the co-dominant (*p* = 0.011) and dominant (*p* = 0.015) models.

## Discussion

Genetic predisposition can influence an individual’s perception of their quality of life [6]. In the field of dentistry, few studies [8–12] have explored this area, and none have addressed polymorphisms in SOD genes in individuals with asymptomatic periapical periodontitis who have undergone root canal treatment. Consequently, our study proposed the hypothesis that genetic polymorphisms in *SOD2* (rs5746136, rs4880, and rs10370) and *SOD3* (rs2855262 and rs13306703) could modulate the impact of OHRQoL in patients with asymptomatic periapical periodontitis. The findings of our study supported this hypothesis, revealing that genetic polymorphisms in the *SOD2* and *SOD3* genes influence the OHRQoL response. The analyses were conducted using the co-dominant, recessive, and dominant genetic models. The decision to conduct these types of analyses stems from the recognition that genetic variations can manifest diverse modes of inheritance. By exploring these different models, researchers gain a comprehensive understanding of how specific genetic factors may influence the phenotype of interest. Co-dominant analysis considers the impact of heterozygous genotypes, while recessive and dominant analyses focus on the effects of homozygous genotypes.

OHRQoL is a multidimensional construct encompassing a subjective evaluation of an individual’s oral health, functional well-being, emotional well-being, expectations, satisfaction with care, and self-perception [40]. Sociodental indicators, the term generically applied to questionnaires designed to assess the impact of oral problems on quality of life, were used in this study [41]. The Brazilian version of the OHIP-14 form was selected to evaluate the impact of polymorphism in the *SOD2* and *SOD3* genes on OHRQoL [37]. The OHIP-14, which includes seven dimensions [42], is a valid, reliable, and widely used tool for assessing quality of life in epidemiological studies [9, 10, 14, 16]. The OHIP-14 was administered in an interview format at three distinct times: before treatment, 7 days post-treatment, and 30 days post-treatment. These factors did not affect our results, as the psychometric properties of the OHIP-14 are not influenced by the mode of application [43]. Furthermore, using a shorter reference period, such as 1 month, does not appear to impact responses [44] and may even reduce memory bias [45].

Periapical periodontitis is a multifaceted condition, influenced by a combination of genetic and environmental factors that affect its susceptibility and progression [46]. SODs, known for their role in oxidative stress, contribute to local periapical tissue damage [21, 22]. This damage results from an imbalance between the overproduction of ROS and the availability of antioxidants [24]. In summary, oxidative stress is central to the pathogenesis of periapical periodontitis. While ROS serve as a crucial defense mechanism against endodontic bacterial challenges and modulate cell signaling, an imbalance of oxidants contributes to the formation and progression of periapical periodontitis. This contribution is through direct molecular damage and redox-signaling [22]. Changes in the redox state are associated with the bone remodeling process, which facilitates continuous bone regeneration through the coordinated action of osteoclasts, osteoblasts, and osteocytes. Consequently, alterations in ROS and/or antioxidant systems may play a role in the pathogenesis of bone loss. ROS induce apoptosis in osteoblasts and osteocytes, promoting osteoclastogenesis and inhibiting mineralization and osteogenesis [25]. ROS are produced during normal metabolism following the activation of various enzymes such as NAD(P)H) oxidase (a membrane enzyme), SOD (a cytoplasmic enzyme), and various mitochondrial oxidases [24, 47, 48]. A controlled increase in ROS levels, particularly H2O2, may play a significant role in transmitting intracellular signals that regulate essential cellular processes such as proliferation, differentiation, apoptosis, repair processes, and inflammation [26, 48].

SODs are a ubiquitous family of enzymes that efficiently catalyze the dismutation of superoxide anions. *SOD2*, also known as Mn-SOD (EC 1.15.1.1), is located on chromosome 6. It exists as a tetramer and is initially synthesized with a leader peptide, which guides this manganese-containing enzyme exclusively to the mitochondrial spaces. *SOD3*, or EC-SOD (EC 1.15.1.1), is located on chromosome 4. This enzyme, characterized more recently, also exists as a tetramer. However, it contains copper and zinc and is synthesized with a signal peptide that directs it exclusively to extracellular spaces [49].

To date, no studies have been conducted to explore the potential association between polymorphism in oxidative stress genes and OHRQoL in patients with asymptomatic periapical periodontitis who have undergone root canal treatment. Oxidative stress caused by reactive species, including ROS and reactive nitrogen species (RNS), is an underlying cause of various neurodegenerative diseases [50]. Animal models of anxiety are accompanied by multiple indices of nitro-oxidative stress including elevated generation of ROS and RNS, including nitric oxide (NO) production; increased lipid peroxidation with the formation of aldehydes, such as malondialdehyde; and lowered antioxidant enzymes including catalase and glutathione peroxidase. These findings suggest the hippocampus might be one of the prime brain structures involved in this state of oxidative stress imbalance [50–52]. ROS and RNS are normally generated by tightly regulated enzymes, such as NO synthase (NOS) and NAD(P)H oxidase isoforms, respectively. Overproduction of ROS (arising either from a mitochondrial electron-transport chain or excessive stimulation of NAD(P)H) results in oxidative stress, a deleterious process that can be an important mediator of damage to cell structures, including lipids and membranes, proteins, and DNA [24].

Our findings suggest that genetic polymorphism in *SOD2* and *SOD3* genes may influence the impact on OHRQoL in these patients, primarily in the domains of functional limitation, physical pain, psychological discomfort, psychological disability, handicap, and total score. The GeneQoL Consortium posits that the inflammatory pathway presents the most compelling evidence as a mechanism for managing underlying fatigue. Furthermore, inflammation and neurotransmission are crucial processes in pain perception [6], which may elucidate this outcome. Moreover, alterations in SODs may be implicated in psychological disorders [28, 29], as explained above. Therefore, further research involving diverse populations and oral conditions is necessary to enhance our understanding of the role of SODs in quality of life.

The current study has multiple strengths, primarily owing to its methodologically sound design. This includes the inclusion of a sample with clearly defined criteria and the successful extraction of DNA following a protocol previously established in the literature. The diagnostic use of saliva offers several benefits: it is easily collected and employs a non-invasive technique. It can also be utilized to measure oxidative stress markers, which are associated with local oral conditions [53]. Furthermore, a validated instrument was employed at specific intervals, thereby minimizing memory bias and the patient’s perception of the procedure. However, it is crucial to acknowledge certain limitations. Firstly, despite the proven reliability of the OHIP-14, there is a potential for information bias. Secondly, the Hawthorne effect may be present, where research participants may consciously alter their behavior when aware of being observed and monitored, leading to an overestimation of the intervention’s effectiveness. Lastly, the potential for a Type 1 error exists, where a true null hypothesis might be mistakenly rejected. Despite the implementation of statistical strategies to mitigate this risk, the complete elimination of this possibility remains challenging. Concerning Type 2 error, there is apprehension about the chance of not rejecting a false null hypothesis. Uncontrolled variables or overlooked factors may influence the results, contributing to the occurrence of this error type. It is crucial to maintain a critical perspective when interpreting the results, taking these limitations into account for a more accurate and robust assessment of the study’s conclusions. Future studies are necessary to either confirm or refute the findings of this study.

The multifaceted nature of OHRQoL is beneficial in identifying at-risk populations and formulating interventions that cater to the ‘whole person’ [40]. Consequently, this study serves as an initial step toward exploring the intricate relationship between genetic polymorphisms and OHRQoL in patients with asymptomatic periapical periodontitis undergoing root canal treatment. Moreover, a deeper understanding of the mechanisms regulating cell and tissue-specific SOD gene expression and their signal transduction pathways could potentially pave the way for the development of novel drugs and strategies. These would aim to regulate the levels of these enzymes in specific tissues, cell types, and compartments without impacting other cells [49]. This would provide differentiated treatment protocols based on the analysis of genetic aspects.

## Conclusions

Polymorphisms in the *SOD2* and *SOD3* genes influenced the OHRQoL of patients with asymptomatic periapical periodontitis who are undergoing root canal treatment.

## Supplementary Material

Is there an association between superoxide dismutase gene polymorphisms, antioxidants in oxidative stress pathway, and oral health-related quality of life after root canal treatment?
