# Supplementary material for: Is there an association between superoxide dismutase gene polymorphisms, antioxidants in oxidative stress pathway, and oral health-related quality of life after root canal treatment?
Source: Acta Odontol Scand. 2025 Apr 22;84:43426. doi: 10.2340/aos.v84.43426 (PMC12056317; doi:10.2340/aos.v84.43426)
Supplement: Is there an association between superoxide dismutase gene polymorphisms, antioxidants in oxidative stress pathway, and oral health-related quality of life after root canal treatment? [file AOS-84-43426-s1.pdf]

Supplementary material has been published as submitted. It has not been copyedited or typeset by Acta Odontologica Scandinavica.

Supplementary Table 1. Impact of genetic polymorphisms on functional limitation of OHIP-14 before, 7 and 30 days after root canal treatment.

				Functional limitation												
Gene	Genetic polymorphism	Model	Genotype	Before treatment				After 7 days				After 30 days				
				No impact		Impact		No impact		Impact		No impact		Impact		
				N	%	N	%	N	%	N	%	N	%	N	%	P value
SOD3	rs2855262	Co-Dominant	TT	18	25.71	13	34.21	28	28.00	3	42.86	30	28.30	1	33.33	Reference
			CT	35	50.00	21	55.26	52	52.00	4	57.14	54	50.94	2	66.67	0.607
			CC	17	24.29	4	10.53	20	20.00	0	0.00	22	20.75	0	0.00	**0.037***
		Dominant	TT	18	25.71	13	34.21	28	28.00	3	42.86	30	28.30	1	33.33	Reference
			CT + CC	52	74.29	25	65.79	72	72.00	4	57.14	76	71.70	2	66.67	0.248
		Recessive	TT + CT	53	75.71	34	89.47	80	80.00	7	100.00	84	79.25	3	100.00	Reference
			CC	17	24.29	4	10.53	20	20.00	0	0.00	22	20.75	0	0.00	**0.040***
	rs13306703	Co-Dominant	CC	53	75.71	29	76.32	78	78.00	3	42.86	80	75.47	3	100.00	Reference
			CT	16	22.86	7	18.42	20	20.00	3	42.86	23	21.70	0	0.00	0.903
			TT	1	1.43	2	5.26	2	2.00	1	14.29	3	2.83	0	0.00	0.082
		Dominant	CC	53	75.71	29	76.32	78	78.00	3	42.86	80	75.47	3	100.00	Reference
			CT + TT	17	24.29	9	23.68	22	22.00	4	57.14	26	24.53	0	0.00	0.530
		Recessive	CC + CT	69	98.57	36	94.74	98	98.00	6	85.71	103	97.17	3	100.00	Reference
			TT	1	1.43	2	5.26	2	2.00	1	14.29	3	2.83	0	0.00	0.080

SOD2	rs5746136	Co-Dominant	CC	35	50.00	25	64.10	58	57.43	2	28.57	58	54.21	2	66.67	Reference
			CT	29	41.43	14	35.90	37	36.63	5	71.43	43	40.19	1	33.33	#
			TT	6	8.57	0	0.00	6	5.94	0	0.00	6	5.61	0	0.00	#
		Dominant	CC	35	50.00	25	64.10	58	57.43	2	28.57	58	54.21	2	66.67	Reference
			CT + TT	35	50.00	14	35.90	43	42.57	5	71.43	49	45.79	1	33.33	0.642
		Recessive	CC + CT	64	91.43	39	100.00	95	94.06	7	100.00	101	94.39	3	100.00	Reference
			TT	6	8.57	0	0.00	6	5.94	0	0.00	6	5.61	0	0.00	#
	rs4880	Co-Dominant	AA	23	32.86	10	26.32	29	29.00	4	57.14	32	30.19	1	33.33	Reference
			AG	34	48.57	15	39.47	47	47.00	1	14.29	49	46.23	1	33.33	0.443
			GG	13	18.57	13	34.21	24	24.00	2	28.57	25	23.58	1	33.33	0.397
		Dominant	AA	23	32.86	10	26.32	29	29.00	4	57.14	32	30.19	1	33.33	Reference
			AG + GG	47	67.14	28	73.68	71	71.00	3	42.86	74	69.81	2	66.67	0.872
		Recessive	AA + AG	57	81.43	25	65.79	76	76.00	5	71.43	81	76.42	2	66.67	Reference
			GG	13	18.57	13	34.21	24	24.00	2	28.57	25	23.58	1	33.33	0.095
	rs10370	Co-Dominant	TT	42	60.00	27	72.97	65	65.66	4	57.14	66	62.86	3	100.00	Reference
			TG	24	34.29	10	27.03	30	30.30	3	42.86	35	33.33	0	0.00	#
			GG	4	5.71	0	0.00	4	4.04	0	0.00	4	3.81	0	0.00	#
		Dominant	TT	42	60.00	27	72.97	65	65.66	4	57.14	66	62.86	3	100.00	Reference
			TG + GG	28	40.00	10	27.03	34	34.34	3	42.86	39	37.14	0	0.00	0.218
		Recessive	TT + TG	66	94.29	37	100.00	95	95.96	7	100.00	101	96.19	3	100.00	Reference
			GG	4	5.71	0	0.00	4	4.04	0	0.00	4	3.81	0	0.00	#

Notes: Univariate Poisson Regression by Generalized Estimating Equations was performed to obtain p-value. # means that some convergence criteria were not met. *means $p < 0.05$.

Supplementary Table 2. Impact of genetic polymorphisms on physical pain of OHIP-14 before, 7 and 30 days after root canal treatment.

				Physical pain													
Gene	Genetic polymorphism	Model	Genotype	Before treatment				After 7 days				After 30 days					
				No impact		Impact		No impact		Impact		No impact		Impact			
				N	%	N	%	N	%	N	%	N	%	N	%	P value	
SOD3	rs2855262	Co-Dominant	TT	9	60.00	22	23.66	21	29.58	10	27.78	25	29.76	6	24.00	Reference	
			CT	4	26.67	52	55.91	34	47.89	22	61.11	42	50.00	14	56.00	0.109	
			CC	2	13.33	19	20.43	16	22.54	4	11.11	17	20.24	5	20.00	0.635	
		Dominant	TT	9	60.00	22	23.66	21	29.58	10	27.78	25	29.76	6	24.00	Reference	
			CT + CC	6	40.00	71	76.34	50	70.42	26	72.22	59	70.24	19	76.00	0.173	
		Recessive	TT + CT	13	86.67	74	79.57	55	77.46	32	88.89	67	79.76	20	80.00	Reference	
			CC	2	13.33	19	20.43	16	22.54	4	11.11	17	20.24	5	20.00	0.488	
	rs13306703	Co-Dominant	CC	10	66.67	72	77.42	53	74.65	28	77.78	63	75.00	20	80.00	Reference	
			CT	5	33.33	18	19.35	18	25.35	5	13.89	19	22.62	4	16.00	0.135	
			TT	0	0.00	3	3.23	0	0.00	3	8.33	2	2.38	1	4.00	**0.001***	
		Dominant	CC	10	66.67	72	77.42	53	74.65	28	77.78	63	75.00	20	80.00	Reference	
			CT + TT	5	33.33	21	22.58	18	25.35	8	22.22	21	25.00	5	20.00	0.404	
		Recessive	CC + CT	15	100.00	90	96.77	71	100.00	33	91.67	82	97.62	24	96.00	Reference	
			TT	0	0.00	3	3.23	0	0.00	3	8.33	2	2.38	1	4.00	**<0.001***	

SOD2	rs5746136	Co-Dominant	CC	8	53.33	52	55.32	36	50.70	24	64.86	46	54.76	14	53.85	Reference
			CT	6	40.00	37	39.36	32	45.07	10	27.03	34	40.48	10	38.46	0.346
			TT	1	6.67	5	5.32	3	4.23	3	8.11	4	4.76	2	7.69	0.632
		Dominant	CC	8	53.33	52	55.32	36	50.70	24	64.86	46	54.76	14	53.85	Reference
			CT + TT	7	46.67	42	44.68	35	49.30	13	35.14	38	45.24	12	46.15	0.482
		Recessive	CC + CT	14	93.33	89	94.68	68	95.77	34	91.89	80	95.24	24	92.31	Reference
			TT	1	6.67	5	5.32	3	4.23	3	8.11	4	4.76	2	7.69	0.494
	rs4880	Co-Dominant	AA	3	20.00	30	32.26	20	28.17	13	36.11	23	27.38	10	40.00	Reference
			AG	8	53.33	41	44.09	36	50.70	12	33.33	40	47.62	10	40.00	0.082
			GG	4	26.67	22	23.66	15	21.13	11	30.56	21	25.00	5	20.00	0.586
		Dominant	AA	3	20.00	30	32.26	20	28.17	13	36.11	23	27.38	10	40.00	Reference
			AG + GG	12	80.00	63	67.74	51	71.83	23	63.89	61	72.62	15	60.00	0.134
		Recessive	AA + AG	11	73.33	71	76.34	56	78.87	25	69.44	63	75.00	20	80.00	Reference
			GG	4	26.67	22	23.66	15	21.13	11	30.56	21	25.00	5	20.00	0.744
	rs10370	Co-Dominant	TT	8	53.33	61	66.30	43	61.43	26	72.22	53	63.86	16	64.00	Reference
			TG	6	40.00	28	30.43	24	34.29	9	25.00	28	33.73	7	28.00	0.255
			GG	1	6.67	3	3.26	3	4.29	1	2.78	2	2.41	2	8.00	0.990
		Dominant	TT	8	53.33	61	66.30	43	61.43	26	72.22	53	63.86	16	64.00	Reference
			TG + GG	7	46.67	31	33.70	27	38.57	10	27.78	30	36.14	9	36.00	0.308
		Recessive	TT + TG	14	93.33	89	96.74	67	95.71	35	97.22	81	97.59	23	92.00	Reference
			GG	1	6.67	3	3.26	3	4.29	1	2.78	2	2.41	2	8.00	0.895

Notes: Univariate Poisson Regression by Generalized Estimating Equations was performed to obtain p-value. # means that some convergence criteria were not met. *means $p < 0.05$.

Supplementary Table 3. Impact of genetic polymorphisms on psychological discomfort of OHIP-14 before, 7 and 30 days after root canal treatment.

				Psychological discomfort												
**Gene**	**Genetic polymorphism**	**Model**	**Genotype**	**Before treatment**				**After 7 days**				**After 30 days**				
				No impact		Impact		No impact		Impact		No impact		Impact		
				N	%	N	%	N	%	N	%	N	%	N	%	P value
SOD3	rs2855262	Co-Dominant	TT	3	27.27	28	28.87	17	25.37	14	35.00	19	25.33	12	35.29	Reference
			CT	4	36.36	52	53.61	37	55.22	19	47.50	39	52.00	17	50.00	0.392
			CC	4	36.36	17	17.53	13	19.40	7	17.50	17	22.67	5	14.71	0.200
		Dominant	TT	3	27.27	28	28.87	17	25.37	14	35.00	19	25.33	12	35.29	Reference
			CT + CC	8	72.73	69	71.13	50	74.63	26	65.00	56	74.67	22	64.71	0.248
		Recessive	TT + CT	7	63.64	80	82.47	54	80.60	33	82.50	58	77.33	29	85.29	Reference
			CC	4	36.36	17	17.53	13	19.40	7	17.50	17	22.67	5	14.71	0.310
	rs13306703	Co-Dominant	CC	9	81.82	73	75.26	53	79.10	28	70.00	57	76.00	26	76.47	Reference
			CT	2	18.18	21	21.65	14	20.90	9	22.50	16	21.33	7	20.59	0.761
			TT	0	0.00	3	3.09	0	0.00	3	7.50	2	2.67	1	2.94	**0.002***
		Dominant	CC	9	81.82	73	75.26	53	79.10	28	70.00	57	76.00	26	76.47	Reference
			CT + TT	2	18.18	24	24.74	14	20.90	12	30.00	18	24.00	8	23.53	0.460
		Recessive	CC + CT	11	100.00	94	96.91	67	100.00	37	92.50	73	97.33	33	97.06	Reference
			TT	0	0.00	3	3.09	0	0.00	3	7.50	2	2.67	1	2.94	**0.002***

SOD2	rs5746136	Co-Dominant	CC	6	54.55	54	55.10	39	57.35	21	52.50	44	57.89	16	47.06	Reference
			CT	3	27.27	40	40.82	24	35.29	18	45.00	28	36.84	16	47.06	0.272
			TT	2	18.18	4	4.08	5	7.35	1	2.50	4	5.26	2	5.88	0.416
		Dominant	CC	6	54.55	54	55.10	39	57.35	21	52.50	44	57.89	16	47.06	Reference
			CT + TT	5	45.45	44	44.90	29	42.65	19	47.50	32	42.11	18	52.94	0.451
		Recessive	CC + CT	9	81.82	94	95.92	63	92.65	39	97.50	72	94.74	32	94.12	Reference
			TT	2	18.18	4	4.08	5	7.35	1	2.50	4	5.26	2	5.88	0.319
	rs4880	Co-Dominant	AA	4	36.36	29	29.90	20	29.85	13	32.50	20	26.67	13	38.24	Reference
			AG	6	54.55	43	44.33	31	46.27	17	42.50	34	45.33	16	47.06	0.598
			GG	1	9.09	25	25.77	16	23.88	10	25.00	21	28.00	5	14.71	0.543
		Dominant	AA	4	36.36	29	29.90	20	29.85	13	32.50	20	26.67	13	38.24	Reference
			AG + GG	7	63.64	68	70.10	47	70.15	27	67.50	55	73.33	21	61.76	0.534
		Recessive	AA + AG	10	90.91	72	74.23	51	76.12	30	75.00	54	72.00	29	85.29	Reference
			GG	1	9.09	25	25.77	16	23.88	10	25.00	21	28.00	5	14.71	0.691
	rs10370	Co-Dominant	TT	6	54.55	63	65.63	42	62.69	27	69.23	49	66.22	20	58.82	Reference
			TG	4	36.36	30	31.25	22	32.84	11	28.21	22	29.73	13	38.24	0.977
			GG	1	9.09	3	3.13	3	4.48	1	2.56	3	4.05	1	2.94	0.472
		Dominant	TT	6	54.55	63	65.63	42	62.69	27	69.23	49	66.22	20	58.82	Reference
			TG + GG	5	45.45	33	34.38	25	37.31	12	30.77	25	33.78	14	41.18	0.833
		Recessive	TT + TG	10	90.91	93	96.88	64	95.52	38	97.44	71	95.95	33	97.06	Reference
			GG	1	9.09	3	3.13	3	4.48	1	2.56	3	4.05	1	2.94	0.472

Notes: Univariate Poisson Regression by Generalized Estimating Equations was performed to obtain p-value. # means that some convergence criteria were not met. *means $p < 0.05$.

Supplementary Table 4. Impact of genetic polymorphisms on physical disability of OHIP-14 before, 7 and 30 days after root canal treatment.

Gene	Genetic polymorphism	Model	Genotype	Physical disability												
				Before treatment				After 7 days				After 30 days				
				No impact		Impact		No impact		Impact		No impact		Impact		
				N	%	N	%	N	%	N	%	N	%	N	%	P value
SOD3	rs2855262	Co-Dominant	TT	16	29.63	15	27.78	24	26.97	7	38.89	26	26.53	5	45.45	Reference
			CT	26	48.15	30	55.56	47	52.81	9	50.00	51	52.04	5	45.45	0.662
			CC	12	22.22	9	16.67	18	20.22	2	11.11	21	21.43	1	9.09	0.214
		Dominant	TT	16	29.63	15	27.78	24	26.97	7	38.89	26	26.53	5	45.45	Reference
			CT + CC	38	70.37	39	72.22	65	73.03	11	61.11	72	73.47	6	54.55	0.434
		Recessive	TT + CT	42	77.78	45	83.33	71	79.78	16	88.89	77	78.57	10	90.91	Reference
			CC	12	22.22	9	16.67	18	20.22	2	11.11	21	21.43	1	9.09	0.243
	rs13306703	Co-Dominant	CC	40	74.07	42	77.78	68	76.40	13	72.22	77	78.57	6	54.55	Reference
			CT	13	24.07	10	18.52	19	21.35	4	22.22	18	18.37	5	45.45	0.709
			TT	1	1.85	2	3.70	2	2.25	1	5.56	3	3.06	0	0.00	0.538
		Dominant	CC	40	74.07	42	77.78	68	76.40	13	72.22	77	78.57	6	54.55	Reference
			CT + TT	14	25.93	12	22.22	21	23.60	5	27.78	21	21.43	5	45.45	0.620
		Recessive	CC + CT	53	98.15	52	96.30	87	97.75	17	94.44	95	96.94	11	100.00	Reference
			TT	1	1.85	2	3.70	2	2.25	1	5.56	3	3.06	0	0.00	0.571

SOD2	rs5746136	Co-Dominant	CC	29	53.70	31	56.36	49	54.44	11	61.11	55	55.56	5	45.45	Reference
			CT	21	38.89	22	40.00	37	41.11	5	27.78	38	38.38	6	54.55	0.963
			TT	4	7.41	2	3.64	4	4.44	2	11.11	6	6.06	0	0.00	0.641
		Dominant	CC	29	53.70	31	56.36	49	54.44	11	61.11	55	55.56	5	45.45	Reference
			CT + TT	25	46.30	24	43.64	41	45.56	7	38.89	44	44.44	6	54.55	0.903
		Recessive	CC + CT	50	92.59	53	96.36	86	95.56	16	88.89	93	93.94	11	100.00	Reference
			TT	4	7.41	2	3.64	4	4.44	2	11.11	6	6.06	0	0.00	0.636
	rs4880	Co-Dominant	AA	15	27.78	18	33.33	26	29.21	7	38.89	28	28.57	5	45.45	Reference
			AG	29	53.70	20	37.04	42	47.19	6	33.33	45	45.92	5	45.45	0.166
			GG	10	18.52	16	29.63	21	23.60	5	27.78	25	25.51	1	9.09	0.764
		Dominant	AA	15	27.78	18	33.33	26	29.21	7	38.89	28	28.57	5	45.45	Reference
			AG + GG	39	72.22	36	66.67	63	70.79	11	61.11	70	71.43	6	54.55	0.250
		Recessive	AA + AG	44	81.48	38	70.37	68	76.40	13	72.22	73	74.49	10	90.91	Reference
			GG	10	18.52	16	29.63	21	23.60	5	27.78	25	25.51	1	9.09	0.611
	rs10370	Co-Dominant	TT	34	62.96	35	66.04	57	64.77	12	66.67	62	63.92	7	63.64	Reference
			TG	18	33.33	16	30.19	27	30.68	6	33.33	31	31.96	4	36.36	0.926
			GG	2	3.70	2	3.77	4	4.55	0	0.00	4	4.12	0	0.00	0.387
		Dominant	TT	34	62.96	35	66.04	57	64.77	12	66.67	62	63.92	7	63.64	Reference
			TG + GG	20	37.04	18	33.96	31	35.23	6	33.33	35	36.08	4	36.36	0.802
		Recessive	TT + TG	52	96.30	51	96.23	84	95.45	18	100.00	93	95.88	11	100.00	Reference
			GG	2	3.70	2	3.77	4	4.55	0	0.00	4	4.12	0	0.00	0.390

Notes: Univariate Poisson Regression by Generalized Estimating Equations was performed to obtain p-value. # means that some convergence criteria were not met. *means $p < 0.05$.

Supplementary Table 5. Impact of genetic polymorphisms on psychological disability of OHIP-14 before, 7 and 30 days after root canal treatment.

Gene	Genetic polymorphism	Model	Genotype	Psychological disability												
				Before treatment				After 7 days				After 30 days				
				No impact		Impact		No impact		Impact		No impact		Impact		
				N	%	N	%	N	%	N	%	N	%	N	%	P value
SOD3	rs2855262	Co-Dominant	TT	6	23.08	25	30.49	23	26.14	8	42.11	26	27.96	5	31.25	Reference
			CT	16	61.54	40	48.78	46	52.27	10	52.63	48	51.61	8	50.00	0.332
			CC	4	15.38	17	20.73	19	21.59	1	5.26	19	20.43	3	18.75	0.310
		Dominant	TT	6	23.08	25	30.49	23	26.14	8	42.11	26	27.96	5	31.25	Reference
			CT + CC	20	76.92	57	69.51	65	73.86	11	57.89	67	72.04	11	68.75	0.269
		Recessive	TT + CT	22	84.62	65	79.27	69	78.41	18	94.74	74	79.57	13	81.25	Reference
			CC	4	15.38	17	20.73	19	21.59	1	5.26	19	20.43	3	18.75	0.566
	rs13306703	Co-Dominant	CC	23	88.46	59	71.95	68	77.27	13	68.42	71	76.34	12	75.00	Reference
			CT	2	7.69	21	25.61	18	20.45	5	26.32	19	20.43	4	25.00	0.112
			TT	1	3.85	2	2.44	2	2.27	1	5.26	3	3.23	0	0.00	0.960
		Dominant	CC	23	88.46	59	71.95	68	77.27	13	68.42	71	76.34	12	75.00	Reference
			CT + TT	3	11.54	23	28.05	20	22.73	6	31.58	22	23.66	4	25.00	0.154
		Recessive	CC + CT	25	96.15	80	97.56	86	97.73	18	94.74	90	96.77	16	100.00	Reference
			TT	1	3.85	2	2.44	2	2.27	1	5.26	3	3.23	0	0.00	0.862

SOD2	rs5746136	Co-Dominant	CC	14	53.85	46	55.42	50	56.18	10	52.63	51	54.26	9	56.25	Reference
			CT	9	34.62	34	40.96	33	37.08	9	47.37	37	39.36	7	43.75	0.655
			TT	3	11.54	3	3.61	6	6.74	0	0.00	6	6.38	0	0.00	0.065
		Dominant	CC	14	53.85	46	55.42	50	56.18	10	52.63	51	54.26	9	56.25	Reference
			CT + TT	12	46.15	37	44.58	39	43.82	9	47.37	43	45.74	7	43.75	0.978
		Recessive	CC + CT	23	88.46	80	96.39	83	93.26	19	100.00	88	93.62	16	100.00	Reference
			TT	3	11.54	3	3.61	6	6.74	0	0.00	6	6.38	0	0.00	0.052
	rs4880	Co-Dominant	AA	6	23.08	27	32.93	27	30.68	6	31.58	26	27.96	7	43.75	Reference
			AG	13	50.00	36	43.90	41	46.59	7	36.84	44	47.31	6	37.50	0.261
			GG	7	26.92	19	23.17	20	22.73	6	31.58	23	24.73	3	18.75	0.566
		Dominant	AA	6	23.08	27	32.93	27	30.68	6	31.58	26	27.96	7	43.75	Reference
			AG + GG	20	76.92	55	67.07	61	69.32	13	68.42	67	72.04	9	56.25	0.291
		Recessive	AA + AG	19	73.08	63	76.83	68	77.27	13	68.42	70	75.27	13	81.25	Reference
			GG	7	26.92	19	23.17	20	22.73	6	31.58	23	24.73	3	18.75	0.978
	rs10370	Co-Dominant	TT	17	65.38	52	64.20	57	65.52	12	63.16	59	64.13	10	62.50	Reference
			TG	7	26.92	27	33.33	26	29.89	7	36.84	29	31.52	6	37.50	0.561
			GG	2	7.69	2	2.47	4	4.60	0	0.00	4	4.35	0	0.00	0.133
		Dominant	TT	17	65.38	52	64.20	57	65.52	12	63.16	59	64.13	10	62.50	Reference
			TG + GG	9	34.62	29	35.80	30	34.48	7	36.84	33	35.87	6	37.50	0.850
		Recessive	TT + TG	24	92.31	79	97.53	83	95.40	19	100.00	88	95.65	16	100.00	Reference
			GG	2	7.69	2	2.47	4	4.60	0	0.00	4	4.35	0	0.00	0.116

Notes: Univariate Poisson Regression by Generalized Estimating Equations was performed to obtain p-value. # means that some convergence criteria were not met. *means p < 0.05.

Supplementary Table 6. Impact of genetic polymorphisms on social disability of OHIP-14 before, 7 and 30 days after root canal treatment.

Social disability																
Gene	Genetic polymorphism	Model	Genotype	Before treatment				After 7 days				After 30 days				
				No impact		Impact		No impact		Impact		No impact		Impact		
				N	%	N	%	N	%	N	%	N	%	N	%	P value
SOD3	rs2855262	Co-Dominant	TT	18	27.69	13	30.23	25	26.32	6	50.00	27	27.27	4	40.00	Reference
			CT	33	50.77	23	53.49	51	53.68	5	41.67	51	51.52	5	50.00	0.416
			CC	14	21.54	7	16.28	19	20.00	1	8.33	21	21.21	1	10.00	0.149
		Dominant	TT	18	27.69	13	30.23	25	26.32	6	50.00	27	27.27	4	40.00	Reference
			CT + CC	47	72.31	30	69.77	70	73.68	6	50.00	72	72.73	6	60.00	0.263
		Recessive	TT + CT	51	78.46	36	83.72	76	80.00	11	91.67	78	78.79	9	90.00	Reference
			CC	14	21.54	7	16.28	19	20.00	1	8.33	21	21.21	1	10.00	0.226
	rs13306703	Co-Dominant	CC	52	80.00	30	69.77	72	75.79	9	75.00	74	74.75	9	90.00	Reference
			CT	12	18.46	11	25.58	21	22.11	2	16.67	22	22.22	1	10.00	0.862
			TT	1	1.54	2	4.65	2	2.11	1	8.33	3	3.03	0	0.00	0.276
		Dominant	CC	52	80.00	30	69.77	72	75.79	9	75.00	74	74.75	9	90.00	Reference
			CT + TT	13	20.00	13	30.23	23	24.21	3	25.00	25	25.25	1	10.00	0.647
		Recessive	CC + CT	64	98.46	41	95.35	93	97.89	11	91.67	96	96.97	10	100.00	Reference
			TT	1	1.54	2	4.65	2	2.11	1	8.33	3	3.03	0	0.00	0.277

SOD2	rs5746136	Co-Dominant	CC	31	47.69	29	65.91	54	56.25	6	50.00	54	54.00	6	60.00	Reference
			CT	29	44.62	14	31.82	36	37.50	6	50.00	40	40.00	4	40.00	0.507
			TT	5	7.69	1	2.27	6	6.25	0	0.00	6	6.00	0	0.00	0.132
		Dominant	CC	31	47.69	29	65.91	54	56.25	6	50.00	54	54.00	6	60.00	Reference
			CT + TT	34	52.31	15	34.09	42	43.75	6	50.00	46	46.00	4	40.00	0.320
		Recessive	CC + CT	60	92.31	43	97.73	90	93.75	12	100.00	94	94.00	10	100.00	Reference
			TT	5	7.69	1	2.27	6	6.25	0	0.00	6	6.00	0	0.00	0.153
	rs4880	Co-Dominant	AA	19	29.23	14	32.56	28	29.47	5	41.67	30	30.30	3	30.00	Reference
			AG	33	50.77	16	37.21	45	47.37	3	25.00	47	47.47	3	30.00	0.225
			GG	13	20.00	13	30.23	22	23.16	4	33.33	22	22.22	4	40.00	0.559
		Dominant	AA	19	29.23	14	32.56	28	29.47	5	41.67	30	30.30	3	30.00	Reference
			AG + GG	46	70.77	29	67.44	67	70.53	7	58.33	69	69.70	7	70.00	0.566
		Recessive	AA + AG	52	80.00	30	69.77	73	76.84	8	66.67	77	77.78	6	60.00	Reference
			GG	13	20.00	13	30.23	22	23.16	4	33.33	22	22.22	4	40.00	0.137
	rs10370	Co-Dominant	TT	38	58.46	31	73.81	61	64.89	8	66.67	61	62.24	8	80.00	Reference
			TG	24	36.92	10	23.81	29	30.85	4	33.33	33	33.67	2	20.00	0.247
			GG	3	4.62	1	2.38	4	4.26	0	0.00	4	4.08	0	0.00	0.254
		Dominant	TT	38	58.46	31	73.81	61	64.89	8	66.67	61	62.24	8	80.00	Reference
			TG + GG	27	41.54	11	26.19	33	35.11	4	33.33	37	37.76	2	20.00	0.175
		Recessive	TT + TG	62	95.38	41	97.62	90	95.74	12	100.00	94	95.92	10	100.00	Reference
			GG	3	4.62	1	2.38	4	4.26	0	0.00	4	4.08	0	0.00	0.307

Notes: Univariate Poisson Regression by Generalized Estimating Equations was performed to obtain p-value. # means that some convergence criteria were not met. *means $p < 0.05$.

Supplementary Table 7. Impact of genetic polymorphisms on handicap of OHIP-14 before, 7 and 30 days after root canal treatment.

Gene	Genetic polymorphism	Model	Genotype	Handicap												
				Before treatment				After 7 days				After 30 days				
				No impact		Impact		No impact		Impact		No impact		Impact		
				N	%	N	%	N	%	N	%	N	%	N	%	P value
SOD3	rs2855262	Co-Dominant	TT	17	27.87	14	29.79	29	28.71	2	33.33	28	26.67	3	75.00	Reference
			CT	28	45.90	28	59.57	53	52.48	3	50.00	55	52.38	1	25.00	0.742
			CC	16	26.23	5	10.64	19	18.81	1	16.67	22	20.95	0	0.00	0.101
		Dominant	TT	17	27.87	14	29.79	29	28.71	2	33.33	28	26.67	3	75.00	Reference
			CT + CC	44	72.13	33	70.21	72	71.29	4	66.67	77	73.33	1	25.00	0.362
		Recessive	TT + CT	45	73.77	42	89.36	82	81.19	5	83.33	83	79.05	4	100.00	Reference
			CC	16	26.23	5	10.64	19	18.81	1	16.67	22	20.95	0	0.00	0.103
	rs13306703	Co-Dominant	CC	52	85.25	30	63.83	76	75.25	5	83.33	81	77.14	2	50.00	Reference
			CT	7	11.48	16	34.04	23	22.77	0	0.00	21	20.00	2	50.00	**0.011***
			TT	2	3.28	1	2.13	2	1.98	1	16.67	3	2.86	0	0.00	0.626
		Dominant	CC	52	85.25	30	63.83	76	75.25	5	83.33	81	77.14	2	50.00	Reference
			CT + TT	9	14.75	17	36.17	25	24.75	1	16.67	24	22.86	2	50.00	**0.015***
		Recessive	CC + CT	59	96.72	46	97.87	99	98.02	5	83.33	102	97.14	4	100.00	Reference
			TT	2	3.28	1	2.13	2	1.98	1	16.67	3	2.86	0	0.00	0.762

SOD2	rs5746136	Co-Dominant	CC	32	51.61	28	59.57	58	56.86	2	33.33	58	54.72	2	50.00	Reference
			CT	25	40.32	18	38.30	38	37.25	4	66.67	42	39.62	2	50.00	0.859
			TT	5	8.06	1	2.13	6	5.88	0	0.00	6	5.66	0	0.00	0.208
		Dominant	CC	32	51.61	28	59.57	58	56.86	2	33.33	58	54.72	2	50.00	Reference
			CT + TT	30	48.39	19	40.43	44	43.14	4	66.67	48	45.28	2	50.00	0.848
		Recessive	CC + CT	57	91.94	46	97.87	96	94.12	6	100.00	100	94.34	4	100.00	Reference
			TT	5	8.06	1	2.13	6	5.88	0	0.00	6	5.66	0	0.00	0.198
	rs4880	Co-Dominant	AA	18	29.51	15	31.91	31	30.69	2	33.33	31	29.52	2	50.00	Reference
			AG	31	50.82	18	38.30	45	44.55	3	50.00	48	45.71	2	50.00	0.473
			GG	12	19.67	14	29.79	25	24.75	1	16.67	26	24.76	0	0.00	0.922
		Dominant	AA	18	29.51	15	31.91	31	30.69	2	33.33	31	29.52	2	50.00	Reference
			AG + GG	43	70.49	32	68.09	70	69.31	4	66.67	74	70.48	2	50.00	0.569
		Recessive	AA + AG	49	80.33	33	70.21	76	75.25	5	83.33	79	75.24	4	100.00	Reference
			GG	12	19.67	14	29.79	25	24.75	1	16.67	26	24.76	0	0.00	0.721
	rs10370	Co-Dominant	TT	36	59.02	33	71.74	66	66.00	3	50.00	66	63.46	3	75.00	Reference
			TG	22	36.07	12	26.09	30	30.00	3	50.00	34	32.69	1	25.00	0.505
			GG	3	4.92	1	2.17	4	4.00	0	0.00	4	3.85	0	0.00	0.352
		Dominant	TT	36	59.02	33	71.74	66	66.00	3	50.00	66	63.46	3	75.00	Reference
			TG + GG	25	40.98	13	28.26	34	34.00	3	50.00	38	36.54	1	25.00	0.385
		Recessive	TT + TG	58	95.08	45	97.83	96	96.00	6	100.00	100	96.15	4	100.00	Reference
			GG	3	4.92	1	2.17	4	4.00	0	0.00	4	3.85	0	0.00	0.385

Notes: Univariate Poisson Regression by Generalized Estimating Equations was performed to obtain p-value. # means that some convergence criteria were not met. *means $p < 0.05$.
